# Effects of shared medical appointments on quality of life and cost-effectiveness for patients with a chronic neuromuscular disease. Study protocol of a randomized controlled trial

**DOI:** 10.1186/1471-2377-11-106

**Published:** 2011-08-23

**Authors:** Femke M Seesing, Gea Drost, Gert-Jan van der Wilt, Baziel GM van Engelen

**Affiliations:** 1 Department of Neurology, Radboud University Nijmegen Medical Center, Reinier Postlaan 4, Nijmegen, The Netherlands; 2 Department of Epidemiology, Biostatistics and Health Technology Assessment, Radboud University Nijmegen Medical Center, Geert Grooteplein noord 21, Nijmegen, The Netherlands

## Abstract

**Background:**

Shared medical appointments are a series of one-to-one doctor-patient contacts, in presence of a group of 6-10 fellow patients. This group visits substitute the annual control visits of patients with the neurologist. The same items attended to in a one-to- one appointment are addressed. The possible advantages of a shared medical appointment could be an added value to the present management of neuromuscular patients. The currently problem-focused one-to-one out-patient visits often leave little time for the patient's psychosocial needs, patient education, and patient empowerment.

**Methods/design:**

A randomized, prospective controlled study (RCT) with a follow up of 6 months will be conducted to evaluate the clinical and cost-effectiveness of shared medical appointments compared to usual care for 300 neuromuscular patients and their partners at the Radboud University Nijmegen Medical Center. Every included patient will be randomly allocated to one of the two study arms. This study has been reviewed and approved by the medical ethics committee of the region Arnhem-Nijmegen, the Netherlands. The primary outcome measure is quality of life as measured by the EQ-5D, SF-36 and the Individualized neuromuscular Quality of Life Questionnaire. The primary analysis will be an intention-to-treat analysis on the area under the curve of the quality of life scores. A linear mixed model will be used with random factor group and fixed factors treatment, baseline score and type of neuromuscular disease. For the economic evaluation an incremental cost-effectiveness analysis will be conducted from a societal perspective, relating differences in costs to difference in health outcome. Results are expected in 2012.

**Discussion:**

This study will be the first randomized controlled trial which evaluates the effect of shared medical appointments versus usual care for neuromuscular patients. This will enable to determine if there is additional value of shared medical appointments to the current therapeutical spectrum. When this study shows that group visits produce the alleged benefits, this may help to increase the acceptance of this innovative and creative way of using one of the most precious resources in health care more efficiently: time.

**Trial registration:**

DutchTrial Register http://www.trialregister.nlNTR1412

## Background

### Introduction

As of January 2006, the department of neurology at the RUNMC has started offering shared medical appointments or group visits to patients with a neuromuscular disease. This novel approach of delivering outpatient care is now being compared with usual care during a randomized controlled trial with 300 patients and their partners. The focus in this trial is on health outcomes and costs.

### Motive

Most neuromuscular diseases are chronic progressive diseases necessitating periodic specialized care. Because of the progressive nature of the disease, existing symptoms aggravate over time and new symptoms may develop over time, requiring adjustment of management, and giving rise to new questions on the part of the patient and his or her partner. In the absence of definitive cures for chronic neuromuscular diseases, the improvement of quality of life, patient- and partner satisfaction with care, self management and functional capacity become key objectives of care. Currently, these patients attend the out-patient clinic at regular intervals (usually annually), where they are seen in one-to-one patient- physician encounters. It is difficult, however, to fulfill the complex needs of neuromuscular patients in these brief, problem-focused out-patient visits which leave little time for the patient's psychosocial needs, patient education, and patient empowerment. The possible advantages of a shared medical appointment could be an answer to these questions. Our hypotheses are that shared medical appointments show 1) the same effect on the development of the disease as individual appointments 2) an improvement of self-efficacy as opposed to individual appointments, therefore resulting in an improved quality of life 3) better use of resource utilization and 4) a positive effect on self efficacy and quality of life of the partner as well as on the relationship [1-6].

### What is a shared medical appointment?

During a shared medical appointment or group visit, 6-10 patients and their partners are seen simultaneously by a physician who is supported by a group mentor. Shared medical appointments are a series of one on one doctor-patient contacts, in presence of a group fellow patients. A group visit takes 1,5 - 2 hours and substitutes the annual control visit of the patients. The same items the neurologist attends to in a one to one appointment are addressed. The physician has more time to give information and patients and partners can ask questions to- and learn from their fellow patients. The group mentor facilitates the group process, fosters interaction between patients and manages time. Shared medical appointments should not be confused with group education or peer support groups. As opposed to a group visit, these meetings do not substitute for the periodic consultation with the clinician [7,8].

Experience with shared medical appointments in patients with diabetes, heart failure, bone marrow transplantation and chronically ill older patients have been reported [9-14]. Evidence of group visits for neurological patients is at this moment at the level of a feasibility trial for patients with Parkinson disease [15].

## Methods/design

**Figure 1:**
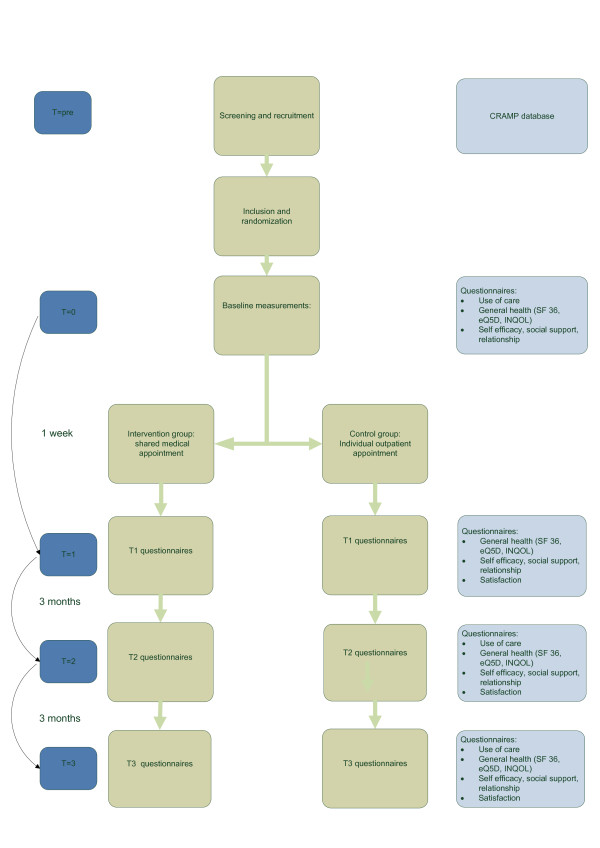
**flowchart of trial design**. CRAMP database: Computer Registry of All Myopathies and Polyneuropathies. INQOL: Individualized Neuromuscular Quality of Life.

A randomized, prospective controlled study (RCT) with a follow up of 6 months will be conducted to evaluate the clinical and cost-effectiveness of shared medical appointments compared to usual care for neuromuscular patients. The trial flow of the proposed subject enrolment and randomization procedures are shown in Figure 1.

### Study population

The aim is to include 270 evaluable patients with one of the following chronic neuromuscular diseases:

- Myotonic Dystrophy type 1 genetically determined (classic and juvenile type) [16];

- McArdles disease, Glycogen Storage Disease Type V. Biochemically and genetically determined;

- Facioscapulohumeral Muscular Dystrophy (FSHD), genetically determined;

- Chronic progressive external ophthalmoplegia (CPEO) as defined by Emery [17];

- Oculopharyngeal Muscular Dystrophy (OPMD), genetically determined;

- Inclusion body myositis (IBM) as defined by Badrising and Verschuuren [18,19]

- Non-dystrophic myotonias. These skeletal muscle channelopathies include two main groups: the chloride and sodium channelopathies [20];

- Myositis: Dermatomyositis and Polymyositis [21]

- Polyneuropathy: CMT1 and HNPP [22]

The partners of these patients will also be included. All patients will be identified by CRAMP, a neuromuscular database containing data from over 4500 patients with neuromuscular disease attending the outpatient clinic of the Radboud University Nijmegen Medical Center [23]. From this database, patients who meet the inclusion criteria (table 1) will be contacted by telephone by the primary investigator (FS) to inform them about the study and ask permission to send information and informed consent forms. If the patient decides not to participate in the study, the reason will be asked and documented. If written consent forms are not returned within 3 weeks, patients will receive a reminder phone call. After written informed consent is obtained, patients and their partners are randomized to one of the two study arms. Reasons for drop-out will be asked and documented.

**Table 1:** inclusion and exclusion criteria

Inclusion criteria	
1.	Registered in CRAMP database with one of the following neuromuscular diseases

	➢ Myotonic Dystrophy type 1. genetically proven (classic and juvenile type)

	➢ McArdles disease, Glycogen Storage Disease Type V. Biochemically and genetically proven

	➢ Facioscapulohumeral Muscular Dystrophy (FSHD). genetically determined

	➢ Chronic progressive external ophthalmoplegia (CPEO) as defined by Emery

	➢ Oculopharyngeal Muscular Dystrophy (OPMD). genetically determined

	➢ Inclusion body myositis (IBM)as defined by Badrising and Verschuuren.

	➢ Non-dystrophic myotonias. These skeletal muscle channelopathies include two main groups: the chloride and sodium channelopathies.

	➢ Myositis: Dermatomyositis and Polymyositis

	➢ Polyneuropathy: CMT1 & HNPP

2.	Age > 18 years

3.	Patients and their partners are control patients in care at the department of neurology RUNMC

**Exclusion criteria**	

1.	Patients or partners with severe hearing problems

2.	Patients or partners who cannot speak, read or understand the Dutch language well

3.	Patients and their partner who have had a control visit with a neurologist at the neurology department of the RUNMC less than 6 months ago

### Ethical approval and registration

This study has been reviewed and approved by the medical ethics committee of the region Arnhem-Nijmegen, the Netherlands. (reference CMO nr. 2008/224) and has been registered in the NTR (Dutch Trial Registration nr NTR1412) Patients and their partners receive verbal and written information about the study and written informed consent will be obtained before randomization.

### Randomization and blinding

Concealed randomization will be performed through computer-generated randomization software. Every included patient will be randomly allocated to one of the two study arms by the computer. The method of simple randomization is applied. In view of the nature of the interventions, blinding of the participants, participating neurologists and primary researcher is not possible. The statistician who conducts the analysis of the data will, however, be blinded for the patients' groups.

### Interventions

Care as usual: individual outpatient appointment.

Patients and partners who are randomized to the control group will have their regular control visit with one of the participating neuromuscular neurologists at the outpatient neurology department of the RUNMC. This one on one control visit substitutes the regular annual control visit the patients pay to the neurology department. The same items that are normally attended to during the control visit, are attended to by the neurologist. All physical examinations, prescriptions and referrals are conducted as deemed appropriate, and documented in the patient record. The regular one on one control visit takes 30 minutes.

Intervention: shared medical appointment

Patients and partners who are randomized to the intervention arm of the study will be invited to a shared medical appointment of 1,5 - 2 hours with one of the participating neuromuscular neurologists and a group mentor at the outpatient neurology department of the RUNMC. The same neurologists execute the individual appointments and the shared medical appointments. As well as for the individual appointments, the shared medical appointments substitute for the annual control visit of the patients. The same items the neurologist attends to in a one on one control visit are attended to during the shared medical appointment. The neurologist is supported by a group mentor, who facilitates the group process, fosters interaction between patients and manages time.

The group mentor starts with a short introduction in which the process of the shared medical appointment is explained and confidentiality is emphasized. Patients and their partners are asked to fill out a privacy form, which is kept in the patient's records, in order to emphasize the confidentiality of the group visit. If physical examinations are deemed necessary, they are conducted in a private examination room close to the group visit room directly after the shared medical appointment. Necessary prescriptions, referrals, and chart notes are being taken care of during or directly after the shared medical appointment. The neurologists have received training in conducting shared medical appointments prior to the study. The process of the shared medical appointments is described in appendix 1.

### Compliance and attrition

When participants indicate that they wish to discontinue their participation in the study, reasons will be documented.

### Outcomes

Outcome measures for patients are listed in table 2. The primary outcome measure is quality of life (daily activity limitations, pain, mood, fatigue, social activities) as measured by the EQ-5D, SF-36 and the Individualized neuromuscular Quality of Life Questionnaire (INQoL). For the SF-36 and EQ 5 D, normative scores are available based on the Dutch population. For the INQoL, no Dutch version was available. With granted authorization from the authors, a translation into the Dutch language was made [24-27]. Secondary outcome measures are (1) Use of health care resources (use of health care services, medication, compliance with medication, use of assistance with daily activities) Questions from the Client Service Receipt Inventory (CSRI) are used to record service utilization [28]. (2) Self efficacy (how much confidence one has in being able to execute specific behaviour), as measured by the Self Efficacy questionnaire from Schwarzer [29]. This is a 10-item psychometric scale that is designed to assess self-beliefs to cope with a variety of difficult demands in life. Also, a questionnaire to measure self efficacy of patients with neuromuscular disease was developed by the research group. (3) The need for social support was assessed by using the subscale emotional support of the Dutch questionnaire SSLD (Sociale Steun Lijst-Discrepanties) from Sanderman and van Sonderen [30]. (4) Satisfaction with relationship was measured by asking the participants to rate their relationship on a 1/10 scale and to rate their relationship on a VAS scale, ranging from the worst possible relationship to the best possible relationship [31] (5) Satisfaction with and aspects of the visit, as measured by The QUality Of care Through the patient's Eyes (QUOTE) questionnaire [32]. The severity of the disease as measured through the Rankin-scale is assessed during the shared medical appointments and through reviewing the chart notes for individual appointments by the participating neurologists or primary researcher [33,34].

**Table 2:** Outcome measures and instrumentation patients

	Instruments	T0	T1	T2	T3
**Primary outcome measures**					

Quality of life	EQ5D, SF36, INQOL	√	√	√	√

**Secondary outcome measures**					

Demographic statistics		√			

Severity of the disease	Rankin Scale	√			

Use of care resources and medicine	Client Service Receipt Inventory (CSRI)	√		√	√

Self efficacy	SE questionnaire from Schwarzer, SE NMD	√	√	√	√

Social support	SSLD (Sociale Steun Lijst-Discrepanties)	√	√	√	√

Satisfaction with relationship		√	√	√	√

Satisfaction with the appointment	QUality Of care Through the patient's Eyes (QUOTE)		√	√	√

Outcome measures for partners are listed in table 3. Among partners of patients, quality of life (EQ-5D) [24-27], self efficacy (SE questionnaire by Schwarzer, [29]), the need for social support (subscale emotional support of the Dutch questionnaire SSLD [30], satisfaction with the relationship (VAS scale and 1-10 reporting)[31], objective burden of care and satisfaction with and aspects of the visit (QUOTE questionnaire, [32])will be measured. Ojective burden of care is measured by asking after the amount of time a partner spends on caring for the partner and on doing household tasks in order to be able to relieve or support their partner with the chronic disease; time spent on delivering care for the chronically ill partner is the main predictor for the impact of the disease on the life of the partner [35].

**Table 3:** Outcome measures and instrumentation partners

	Instruments	T0	T1	T2	T3
**Primary outcome measures**					

Quality of life	EQ5D	√	√	√	√

**Secondary outcome measures**					

Demographic statistics		√			

Self efficacy	SE questionnaire from Schwarzer	√	√	√	√

Social support	SSLD (Sociale Steun Lijst-Discrepanties)	√	√	√	√

Satisfaction with relationship		√	√	√	√

Satisfaction with the appointment	QUality Of care Through the patient's Eyes (QUOTE)		√	√	√

#### Economic evaluation

Cost effectiveness will be assessed from a societal perspective. Direct neuromuscular disease-related costs health care costs, including costs of outpatient care, physical-, speech- and occupational therapy, additional visits to other health care providers (GPs, specialist care, etc.), district nursing, receipt of aids and adaptations, prescription medication, professional home care and hospitalization will be included, as well as non-health care costs such as costs for paid and unpaid help. For unit cost prices, standard rates will be adopted from the national guideline [36] or real cost prices (e.g., for medication) will be obtained through the website of the Dutch Health Care Insurance Board (CVZ, http://www.medicijnkosten.nl). For shared medical appointments, a cost price will be calculated on the basis of available standard rates and real expenditures. The price year will be 2009, the currency Euros, and in view of the timescale of the study, costs and health benefits will not be discounted. Utilization of itemized resources over the trial period will be self-recorded by patients with a modified version of the Client Service Receipt Inventory (CSRI)[28]. Costs per patient will be calculated by multiplying resource volumes by unit costs.

Outcome measures will be obtained through standardized questionnaires, who patients and partners fill out at home at the start of the study period (T0), 1 week (T1), 3 months (T2) and 6 months after the intervention (T3). See Figure 1 and table 2 and 3. At the first measurements (T0) demographic data will be obtained.

### Statistical analysis

#### Sample size

On the basis of the literature [37,38] group visits may be expected to lead to a clinically relevant improvement in quality of life of 5 points on the total SF-36 score. Assuming a standard deviation of 12 [39], 92 patients need to be enrolled in both groups to achieve a statistical power of 80% (alpha = 0.05, two-sided). Taking into account non-evaluable patients, to adjust for imbalances and to be able to do subgroup analysis on age, sex, gender and severity, 135 patients will be enrolled in both groups. In order to be able to evaluate 270 patients, the aim is to include 300 patients.

#### Analysis of outcome measures

300 neuromuscular patients and if applicable their partners will be included in the study. The primary analysis will be an intention-to-treat analysis on the area under the curve of the quality of life scores (average response during 6 months). A linear mixed model will be used with random factor group and fixed factors treatment, baseline score and type of neuromuscular disease. Other outcome parameters will be evaluated in a similar way and additional per protocol analyses will be carried out.

For the analyses SPSS version 17 statistical software will be used. A p-value of 0.05 will be considered statistically significant. Missing data will be imputed using multiple imputation techniques.

#### Economic evaluation

An incremental cost-effectiveness analysis will be conducted from a societal perspective, relating differences in costs to difference in health outcome as measured by the Euroqol-5D, an instrument to evaluate different health states. Bootstrapping will be used for pair-wise comparisons in direct health care costs, direct non-health care costs, total direct costs, total indirect costs, and total costs between the two groups. Confidence intervals will be obtained by conventional re-sampling methods (bootstrapping). The cost effectiveness analysis will provide information on the marginal costs and effects of shared medical appointments relative to conventional one-to-one outpatient visits through the calculation of an incremental cost effectiveness ratio. Ratios will include the primary outcome of the trial, i.e., quality of life and two secondary outcome parameters; functional activities, and self-efficacy. Cost acceptability curves will be calculated showing the probability that the shared medical appointment is cost effective at specified ceiling ratios. In situations where there is no significant difference in effects, the use of cost-minimization analysis will be used for the reporting of cost differences only. Sensitivity analyses will be conducted, exploring the sensitivity of the conclusions to various sources of uncertainty, including sampling variation (e.g., differences in self-efficacy and functional abilities in both groups) and point-estimates (e.g., unit cost prices of major cost drivers).

## Discussion

To the best of our knowledge, this study will be the first randomized controlled trial which evaluates the effect of shared medical appointments versus usual care for neuromuscular patients. Evidence shows that shared medical appointments can have substantial added value, deriving not only from sharing a health care professional's time, but also from sharing mutual experiences, particularly for patients with a chronic disease [2,3,40]. In the literature, group visits have been shown to result in fewer hospitalizations and emergency visits, increased patient satisfaction and increased self-efficacy as compared to usual one-to-one outpatient visits in elderly, chronically ill patients (Scott et al. 2004). In patients with diabetes, a randomized controlled trial demonstrated an increased frequency of preventive procedures among patients attending group visits, resulting in a better general health status as measured by the SF-36 [3]. Sadur et al (1999) demonstrated greater satisfaction with diabetes care, greater self-efficacy, better glycemic control, and lower service utilization among patients with diabetes who were randomly allocated to group visits as compared to counterparts who were allocated to usual care.

This study has several strengths. Firstly, shared medical appointments will be compared with usual care in a randomized design. This will enable to determine if there is additional value of shared medical appointments to the current management spectrum. Secondly, this study involves measuring effect of a treatment on partners of patients with a neuromuscular disease. Studies from Baanders et al (2007) and Timman (2010) show that living with a chronically ill person, specifically with a neuromuscular disorder, has an impact on the partner's life that goes beyond the consequences of care giving, for example consequences on personal life strain, social relations, financial burden, and intrinsic rewards [35]. And that marital satisfaction is a strong predictor of better wellbeing, both for patients and, even more so, for partners [31]. Thirdly, this study aims to determine the cost effectiveness of shared medical appointments as compared to care as usual. In current healthcare time is a scarce good. Efficiency goals are on top of every healthcare managers' list. When introducing a new way of delivering care, such as shared medical appointments, this preferably is just as or even more efficient than care as usual. Therefore it is important to take cost effectiveness of the intervention into account in this study. A limitation of this study can be the fact that it is a single center study. This may influence the transferability of the research results to other hospitals. Due to pragmatic and financial reasons the follow up time is limited to 6 months. For patients with neuromuscular disorders annual control visits are offered, this limits the number of shared medical appointments a patient receives during the study to one. Possibly the effect of group visits increase when attended several times. With this study design it is not possible to show this effect. A reflection of practice in this study, that could be a possible limitation, is inclusion of different patient groups. Although patients all have a chronic neuromuscular disease, possible differences in effects between patient groups could be difficult to show with this study design.

In conclusion, this study will provide greater insight in the (cost) effectiveness of shared medical appointments for neuromuscular patients. The concept of group visits or shared medical appointments is a typical example of organizing the delivery of health care in a different way, in an attempt to improve patient outcome within the limits of available resources. Many attempts at increasing efficiency of health care consist of reducing the amount of time health care professionals spend on specific activities. Such attempts risk, however, to jeopardize the quality of care [41]. When our study shows that group visits produce the alleged benefits, this may help to increase the acceptance of this innovative and creative way of using one of the most precious resources in health care more efficiently: time.

## List of abbreviations used

CPEO:Chronic progressive external ophthalmoplegia; CRAMP database: Computer Registry of All Myopathies and Polyneuropathies; FSHD: Facioscapulohumeral Muscular Dystrophy; IBM: Inclusion body myositis; INQOL: Individualized Neuromuscular Quality of Life; OPMD: Oculopharyngeal Muscular Dystrophy; SMA: shared medical appointment; RCT: randomized controlled trial; RUNMC: Radboud University Nijmegen Medical Center

## Competing interests

The authors declare that they have no competing interests.

## Authors' contributions

FS is primary investigator and responsible for data collection and analysis and for drafting the manuscript. BvE, GJvdW and GD designed and supervised the study. BvE and GJvdW obtained funding for the study. All authors have read and approved the final manuscript.

## Appendix 1

Process of a shared medical appointment:

- Patient registers at the outpatient clinic an led to the group visit room by the group mentor

- Patients are being asked to fill out a privacy form

- Group mentor starts with a brief introduction, in which the process of an SMA and privacy aspects are explained

- Neurologist starts with consulting the first patient

- Individual medical needs and questions of the patient are being discussed with the neurologist

- The neurologist gives information to the patient and his/her partner

- If applicable, the group mentor asks if fellow patients have experiences on this subject they want to share or questions they want to ask to fellow patients or the neurologist

- Neurologist finishes consultation with the first patient and writes notes in the patient record

- The neurologist lifts the second patient record and starts with consulting the second patient, and so on till all patients have had their consultation

- The group mentor asks if all questions are answered and if so closes the group visit

- If applicable, physical examination of a patient is being executed by the neurologist in a separate room, any necessary receipts or blood samples are being taken care of as well

- Group mentor and neurologist leave the group visit room and fill out patient records and patient letters

## Pre-publication history

The pre-publication history for this paper can be accessed here:

http://www.biomedcentral.com/1471-2377/11/106/prepub
